# Real-world evidence on infection risk in multiple myeloma treated with BiTEs and CAR-T cells: a meta-analysis

**DOI:** 10.1186/s40164-026-00798-w

**Published:** 2026-06-20

**Authors:** Federico Spataro, Vanessa Desantis, Giuseppe Dicuonzo, Stefano Molica, Hermann Einsele, Angelo Vacca, Roberto Ria, Antonio Giovanni Solimando

**Affiliations:** 1 Department of Precision and Regenerative Medicine and Ionian Area - DiMePRe-J, Section of Pharmacology, University of Bari Aldo Moro, Bari, 70124 Italy; 2 Department of Precision and Regenerative Medicine and Ionian Area - DiMePRe-J, Guido Baccelli Unit of Internal Medicine, School of Medicine, University of Bari Aldo Moro, Bari, 70124 Italy; 3 Hull York Medical School, University of Hull, Hull, UK; 4 Department of Internal Medicine II, University Hospital, Wurzburg, Germany

**Keywords:** Multiple myeloma, Bispecific antibody, BiTEs, CAR-T, Infection

## Abstract

**Background:**

T-cell-redirecting therapies, including bispecific T-cell engagers (BiTEs) and chimeric antigen receptor T-cell (CAR-T) therapies, have substantially improved outcomes in relapsed or refractory multiple myeloma (RRMM). However, infectious complications remain a major safety concern, particularly in real-world settings, where patients are more heterogeneous than those enrolled in clinical trials.

**Methods:**

We conducted a systematic review and meta-analysis of real-world retrospective studies evaluating severe (grade 3–4) infections in adult patients with RRMM treated with approved BiTEs or CAR-T cell therapies. Pooled event rates were estimated using random-effects models. Heterogeneity was explored through subgroup analyses, meta-regression, and sensitivity analyses.

**Results:**

Sixteen studies encompassing 2,097 patients were included. Overall, 24.2% of patients developed grade 3–4 infections (pooled event rate 0.24; 95% CI, 0.21–0.28). Among BiTEs-treated patients (*n* = 1,602), the pooled severe infection rate was 0.26 (95% CI, 0.23–0.30), with higher rates observed for BCMA-directed BiTEs (0.27) compared with GPRC5D-directed BiTEs (0.25). CAR-T cell therapies (*n* = 495) were associated with a lower pooled infection rate (0.19; 95% CI, 0.12–0.27).

**Conclusions:**

In real-world practice, severe infections affect approximately one in four patients receiving T-cell-redirecting therapies for RRMM. Observed differences in infection rates across platforms and targets should be interpreted with caution, as they derive from indirect comparisons in non-randomized, heterogeneous cohorts. Nevertheless, these data support incorporating patient frailty and prior infection history into therapeutic decision-making. CAR-T therapy, or GPRC5D-directed BiTEs when CAR-T is not feasible, may represent reasonable options in patients at higher infectious risk, within an individualized and context-dependent treatment strategy.

**Supplementary Information:**

The online version contains supplementary material available at 10.1186/s40164-026-00798-w.

**To the Editor,**

T-cell-redirecting immunotherapies, including bispecific antibodies (BiTEs) and chimeric antigen receptor T-cell (CAR-T) therapies, have substantially improved outcomes in relapsed/refractory multiple myeloma (RRMM) [1]. However, infectious complications remain a major safety concern, particularly in real-world settings, where patients are more heterogeneous and clinically vulnerable than those enrolled in prospective clinical trials. While severe infections have been extensively described in pivotal studies, comparative real-world evidence across approved T-cell-redirecting platforms remains limited [2]. 

We conducted a systematic review and meta-analysis of retrospective real-world studies, according to the Preferred Reporting Items for Systematic Reviews and Meta-Analyses (PRISMA) guidelines, evaluating severe (grade 3–4) infections in adult patients with RRMM treated with approved BiTEs or CAR-T therapies, from inception through November 30, 2025 [3]. Detailed Methods, study selection, risk-of-bias assessment and certainty of evidence evaluation are provided in the Supplemental Appendix (S-Figure 1, S-Table 1 and S-Table 2).

Sixteen retrospective studies involving 2,097 patients were included, of whom 1,602 received BiTEs and 495 CAR-T therapies. Mean patient age was 66.3 years, 44.2% were female, and the weighted median follow-up was 8.3 months. Patients were heavily pretreated, with a mean of 6.1 prior lines of therapy. Prior autologous stem cell transplantation was reported in 77.4% of evaluable patients, while extramedullary disease, high-risk cytogenetics, and prior BCMA exposure were present in 39.2%, 48.8%, and 37.0% of evaluable patients, respectively. Additional baseline characteristics are summarized in S-Table 3 and S-Table 4 in the Supplemental Appendix.

Overall, 24.2% of patients developed grade 3–4 infections, corresponding to a pooled event rate of 0.24 (95% confidence interval [CI], 0.21–0.28) (Fig. 1A). Severe infections occurred more frequently in patients treated with BiTEs (pooled event rate 0.26; 95% CI, 0.23–0.30) than in those receiving CAR-T therapies (pooled event rate 0.19; 95% CI, 0.12–0.27). Within the BiTEs cohort, predefined subgroup analyses according to target antigen demonstrated pooled infection rates of 0.27 (95% CI, 0.23–0.31) for BCMA-directed BiTEs and 0.25 (95% CI, 0.20–0.30) for GPRC5D-directed BiTEs (Fig. 1B).

**Fig. 1:**
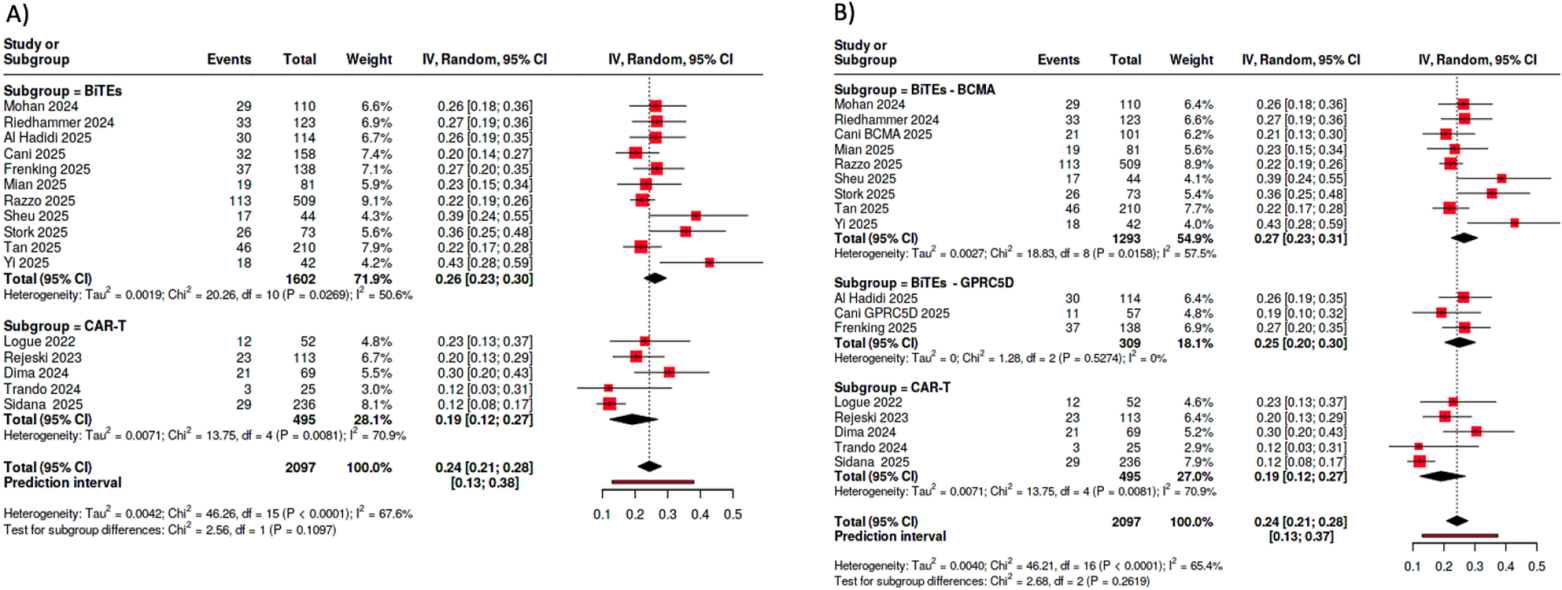
Pooled incidence of grade 3–4 infections in patients with multiple myeloma treated with T-cell-redirecting therapies. **A** Forest plot of pooled event rates of grade 3–4 infections across included real-world studies in patients with multiple myeloma treated with BiTEs and CAR-T cell therapies. **B** Subgroup meta-analysis of pooled grade 3–4 infection rates in BiTEs-treated patients according to target antigen (BCMA-directed vs. GPRC5D-directed). Effect estimates are expressed as pooled proportions with corresponding 95% confidence intervals (CIs) using a random-effects model

Because moderate-to-high heterogeneity was observed, exploratory analyses were performed. In the BiTEs cohort, meta-regression identified an inverse association between severe infection rates and prior BCMA-directed therapy exposure (S-Figure 2), likely reflecting selection bias. Additional BiTEs- and CAR-T-related toxicities, ORR, and available immunoglobulin replacement treatment (IgRT) data are summarized in S-Table 5.

To contextualize our findings, we compared them with previously published meta-analysis on infectious complications associated with BiTEs and CAR-T therapies in RRMM [2, 4–8]. Most available analyses included prospective trials, investigational agents, or mixed study designs. Across these studies, severe infection rates ranged from approximately 20% to 40% for BiTEs and from 17% to 25% for CAR-T therapies, supporting the consistency of our estimates (S-Table 6). BCMA-directed BiTEs showed numerically higher pooled infection rates compared with GPRC5D-directed BiTEs, while CAR-T therapies appeared associated with lower severe infectious burden overall. Although based on indirect comparisons across heterogeneous retrospective cohorts, these findings suggest that infectious toxicity may represent an additional dimension to consider when selecting T-cell-redirecting therapies in routine practice, particularly in patients with frailty, cumulative immunosuppression, cytopenias, or prior severe infections [9]. Notably, emerging evidence suggests that currently available frailty models may incompletely predict infectious vulnerability, further supporting the need for infection-oriented clinical stratification approaches [10]. 

On this basis, we propose a pragmatic conceptual framework integrating infectious vulnerability into therapeutic decision-making (Fig. 2). In clinically fit patients without major infectious history, either CAR-T or BiTEs therapy may represent reasonable options according to availability and clinical context. Conversely, in frail or immunologically vulnerable patients, CAR-T therapy, when feasible, or alternatively GPRC5D-directed BiTEs, may represent potentially safer strategies from an infectious standpoint.

**Fig. 2:**
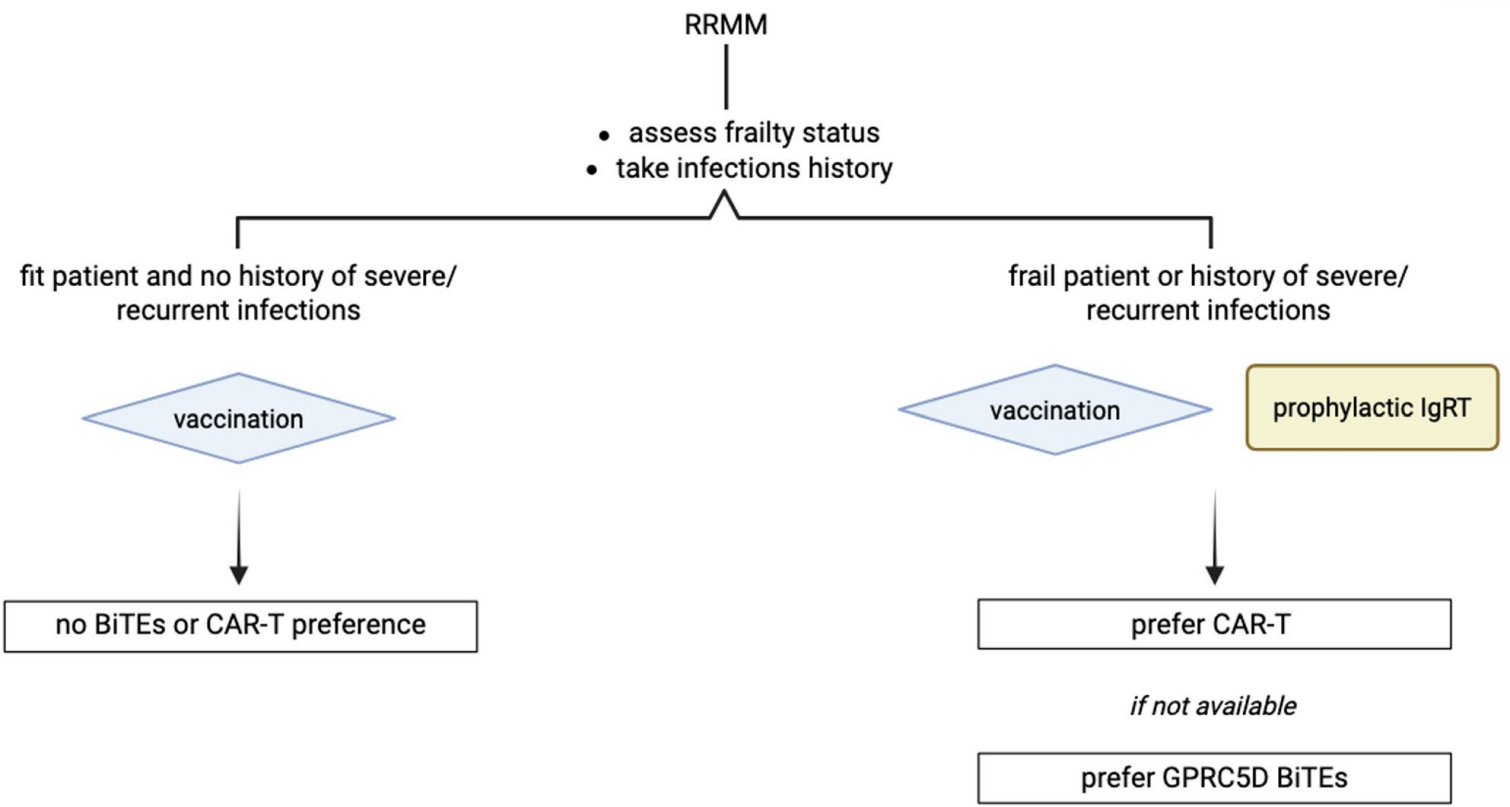
Proposed conceptual framework for interpreting infectious risk across T-cell-redirecting therapies in relapsed or refractory multiple myeloma. This figure illustrates a pragmatic and conceptual framework integrating real-world infection risk to support treatment selection between BiTEs and CAR-T cell therapies in patients with relapsed or refractory multiple myeloma (RRMM). Assessment of patient frailty and prior history of severe or recurrent infections represents the first step in the decision-making process. In patients who are clinically fit and without a history of significant infectious complications, either T-cell-redirecting strategy may be considered appropriate, based on availability and clinical judgment. Conversely, in frail patients or in those with a documented history of severe or recurrent infections, CAR-T cell therapy may be preferentially considered when feasible. When CAR-T therapy is not available or not suitable, GPRC5D-directed BiTEs may represent a potentially safer alternative compared with BCMA-directed BiTEs. In all scenarios, appropriate vaccination strategies are recommended, while immunoglobulin replacement treatment (IgRT) could be taken in consideration for the frailest group. RRMM: relapsed or refractory multiple myeloma. IgRT: immunoglobulin replacement treatment

Our study has limitations inherent to retrospective real-world analyses, including moderate heterogeneity across cohorts, potential patient overlaps among multicenter consortium-based studies, and indirect comparisons between therapeutic platforms. In addition, supportive care variables, including IgRT, antimicrobial prophylaxis, and vaccination strategies, were inconsistently reported and could not be formally evaluated.

Overall, our findings underscore that severe infections remain a major and insufficiently standardized complication of T-cell-redirecting therapies in RRMM, affecting approximately one in four treated patients in real-world practice. Prospective registries with harmonized reporting of infections, antimicrobial prophylaxis, vaccination status, IgRT exposure, frailty-related variables, and infection-related mortality are urgently needed to refine patient selection and develop individualized supportive care strategies.

## Supplementary Information

Below is the link to the electronic supplementary material.

Supplementary Material 1.

## Data Availability

No datasets were generated or analysed during the current study.

## Abbreviations

BCMA: B-cell maturation antigen
BiTEs: Bi-specific T-cell engager or bispecific antibodies
CAR-T: Chimeric antigen receptor T-cell
GPRC5D: G protein-coupled receptor family C group 5 member D
IgRT: Immunoglobulin replacement therapy
MM: Multiple myeloma
PRISMA: Preferred Reporting Items for Systematic Reviews and Meta-Analyses
RRMM: Relapsed or refractory multiple myeloma

## References

[1] Raje NS, Anaissie E, Kumar SK, et al. Consensus guidelines and recommendations for infection prevention in multiple myeloma: a report from the International Myeloma Working Group. Lancet Haematol. 2022;9:e143–61. 35114152 10.1016/S2352-3026(21)00283-0

[2] Vandenboom H, Akhtar O, Szabo A, et al. Safety and efficacy of BCMA CAR-T vs. bispecific antibodies in patients with relapsed multiple myeloma: a systematic review and meta-analysis. Haematologica. 2025. 10.3324/haematol.2025.288174. 40820831 10.3324/haematol.2025.288174 PMC12862333

[3] Page MJ, McKenzie JE, Bossuyt PM, Boutron I, Hoffmann TC, Mulrow CD, et al. The PRISMA 2020 statement: an updated guideline for reporting systematic reviews. BMJ. 2021. 10.1136/bmj.n71. 33782057 10.1136/bmj.n71 PMC8005924

[4] Reynolds G, Cliff ERS, Mohyuddin GR, et al. Infections following bispecific antibodies in myeloma: a systematic review and meta-analysis. Blood Adv. 2023;7:5898–903. 37467036 10.1182/bloodadvances.2023010539 PMC10558589

[5] Wang X, Zhao A, Zhu J, et al. Efficacy and safety of bispecific antibodies therapy for relapsed or refractory multiple myeloma: a systematic review and meta-analysis of prospective clinical trials. Front Immunol. 2024;15:1348955. 38482019 10.3389/fimmu.2024.1348955 PMC10933024

[6] Li W, Zhao D, Jiao Y, et al. Effectiveness and safety of teclistamab for relapsed or refractory multiple myeloma: a systematic review and meta-analysis. Front Immunol. 2025;16:1565407. 40352937 10.3389/fimmu.2025.1565407 PMC12061972

[7] Bakogeorgou S, Filippatos C, Malandrakis P, et al. Safety and Efficacy of Bispecific Antibody Treatment in Relapsed/Refractory Multiple Myeloma: A Systematic Review and Meta-Analysis of Proportions from Clinical Trials. Cancers (Basel). 2025;17:2727. 40940825 10.3390/cancers17172727 PMC12427194

[8] Techaapornkun P, Rojpalakorn W, Mejun N, et al. Comparative efficacy and safety of BCMA-targeted CAR T cells and BiTEs in relapsed/refractory multiple myeloma: a meta-analysis of interventional and real-world studies. Ann Hematol. 2025;104:4791–809. 40924178 10.1007/s00277-025-06524-6 PMC12552310

[9] Rodriguez-Otero P, Usmani S, Cohen AD, et al. International Myeloma Working Group immunotherapy committee consensus guidelines and recommendations for optimal use of T-cell-engaging bispecific antibodies in multiple myeloma. Lancet Oncol. 2024;25:e205–16. 38697166 10.1016/S1470-2045(24)00043-3

[10] Spataro F, Armentaro G, Di Gioia G. Impact of frailty on infection risk in non-transplant eligible multiple myeloma patients: a systematic review and meta-analysis. Leukemia. 2026;40:1072–5. 10.1038/s41375-026-02880-y. 41703031 10.1038/s41375-026-02880-y PMC13149299

