# Supplementary material for: Real-world evidence on infection risk in multiple myeloma treated with BiTEs and CAR-T cells: a meta-analysis
Source: Exp Hematol Oncol. 2026 Jun 20;15:56. doi: 10.1186/s40164-026-00798-w (PMC13282886; doi:10.1186/s40164-026-00798-w)
Supplement: Supplementary file 1 — Supplementary Material 1. [file 40164_2026_798_MOESM1_ESM.docx]

**Methods**

**Search strategy and selection criteria.** This systematic review and meta-analysis were performed and reported according to the Preferred Reporting Items for Systematic Reviews and Meta-Analyses (PRISMA) guidelines (PMID: 33782057). The study protocol was prospectively registered in the PROSPERO database (registration ID: CRD420251163450). A comprehensive literature search was performed in the MEDLINE and LILACS databases from their inception through November 30^th^, 2025, to identify studies reporting severe infection outcomes in patients with RRMM treated with BiTEs or CAR-T cell therapies. The detailed search strategy is provided in S-Figure 1. Eligible studies were required to meet the following criteria: (1) retrospective study design; (2) inclusion of adult patients diagnosed with MM who received BiTEs or CAR-T cell therapy; (3) use of BiTEs or CAR-T products approved for clinical use at the time of the search; and (4) availability of data on the incidence of grade 3-4 infections. Studies evaluating investigational or non-approved products, as well as those without sufficient data for outcome extraction, were excluded. No limitations were imposed with respect to language or year of publication. Additionally, reference lists of included articles, their citing publications, and relevant review articles were manually reviewed to identify further eligible studies. Only retrospective studies were considered to enhance methodological consistency and comparability across datasets. At present, the majority of real-world evidence concerning BiTEs and CAR-T cell therapies in MM originates from retrospective analyses; therefore, restricting inclusion to this study design reduced heterogeneity related to study conduct and outcome assessment.

**Data collection process.** Titles and abstracts were first screened, after which potentially relevant articles underwent full-text evaluation. Data extraction, along with independent appraisal of study quality and risk of bias, was performed by two reviewers (FS and AGS) using the web-based platform Rayyan (PMID: 27919275). Disagreements were resolved through discussion until consensus was reached. For each study meeting the inclusion criteria, information was collected on study design, methodological characteristics, clinical context, eligibility criteria, patient demographics, type of intervention, and reported outcomes.

**Outcomes.** The main outcome evaluated was the proportion of patients with RRMM who experienced grade 3-4 infectious events (severe infections) while receiving BiTEs or CAR-T cell therapy (PMID: 32891586). As all included studies reported infection occurrences in relation to the total number of treated patients, results were summarized using pooled event rates rather than comparative effect estimates.

**Data analysis and risk of bias assessment.** All statistical analyses were performed using the MetanalysisOnline software (PMID: 39928123). Statistical significance was defined by a two-sided p-value of less than 0.05. Between-study variability was quantified using the *I^2^* statistic, which estimates the proportion of total variance attributable to heterogeneity rather than random error (PMID: 38502022). A random-effects model was employed to calculate the pooled incidence of events along with corresponding 95% confidence intervals (CIs). Summary of findings tables were generated using the GRADEpro GDT platform (available at gradepro.org). Study quality was evaluated with the Quality Appraisal of Case Series Studies Checklist developed by the Institute of Health Economics (IHE). Each item was classified as “yes,” “unclear/partial,” or “no,” and studies were considered to have acceptable methodological quality (low to moderate risk of bias) when at least 70% of the criteria were met (https://ihe.ca/document/ihe-quality-appraisal-checklist-for-case-series-studies-with-instructions-for-use/).

Potential publication bias was assessed by visual inspection of funnel plots (PMID: 9310563). The overall certainty of the evidence was rated using the GRADE framework (<https://gdt.gradepro.org/app/handbook/handbook.html>). Meta-regression analyses were undertaken to investigate potential sources of heterogeneity and to assess whether study-level factors were associated with the incidence of severe (grade 3-4) infections. Prespecified moderators included median treatment duration, patient age, sex distribution, prior autologous stem cell transplantation (ASCT), prevalence of extramedullary disease (EMD), high-risk cytogenetic features, International Staging System (ISS) stage I and III, previous exposure to BCMA-directed therapies, and pentarefractory disease status. To preserve statistical robustness and minimize bias related to sparse data, only covariates reported in a sufficient number of studies were included in the meta-regression models. To evaluate the stability of the pooled estimates, a leave-one-out sensitivity analysis was performed by sequentially excluding each study. Between-study heterogeneity was additionally examined using the chi-square (χ²) test and summarized using the *I^2^* statistic (PMID: 12111919).

S-Figure 1. Flow diagram of research screening.

**Identification of studies via databases and registers**

Records removed *before screening*:

- Duplicate records removed (n = 80)
- Records marked as ineligible by automation tools (n = 0)
- Records removed for other reasons (n = 0)

Records identified from:

Medline (n = 86)

LILACS (n= 96)

Total = 182

**Identification**

Records excluded (n = 86):

- Background article (n = 11)
- Publication type not of interest (n = 58)
- Population not of interest (n= 10)
- Outcome not of interest (n = 7)

Records screened

(n = 102)

Reports not retrieved (n = 0)

Reports sought for retrieval

(n = 16)

**Screening**

Reports excluded (n = 0):

Reports assessed for eligibility

(n = 16)

Studies included in review

(n = 16)

**Included**

Medline: ((((multiple myeloma[Title/Abstract]) OR (myeloma[Title/Abstract])) AND ((((((((((bispecific antibod*[Title/Abstract]) OR (BiTEs[Title/Abstract])) OR (elranatamab[Title/Abstract])) OR (teclistamab[Title/Abstract])) OR (talquetamab[Title/Abstract])) OR (CAR-T[Title/Abstract])) OR (idecabtagene[Title/Abstract])) OR (idecel[Title/Abstract])) OR (ciltacabtagene[Title/Abstract])) OR (ciltacel[Title/Abstract]))) AND (infection[Text Word])) NOT (review[Publication Type])) NOT (lymphoma[Title])

**#1 Multiple myeloma** [Ti/Ab] OR myeloma [Ti/Ab] - #2 bispecific [Ti/Ab] OR bites [Ti/Ab] OR elranatamab [Ti/Ab] OR teclistamab [Ti/Ab] OR talquetamab [Ti/Ab] OR CAR-T [Ti/Ab] OR idecabtagene [Ti/Ab] OR ciltacabtagene [Ti/Ab] - #3 infection [TW] - #4 review [PT] - #5 lymphoma [Ti]. #1 AND #2 AND #3 NOT #4 NOT #5.

**LILACS:** (ti:(myeloma)) AND (ti:(bispecific )) OR (ti:(car-t)) AND (safety)

S-Table 1. Quality assessment of the studies.

| Study | **1** | **2** | **3** | **4** | **5** | **6** | **7** | **8** | **9** | **10** | **11** | **12** | **13** | **14** | **15** | **16** | **17** | **18** | **19** | **20** | **Quality score** |
| --- | --- | --- | --- | --- | --- | --- | --- | --- | --- | --- | --- | --- | --- | --- | --- | --- | --- | --- | --- | --- | --- |
| ***BiTEs*** |  |  |  |  |  |  |  |  |  |  |  |  |  |  |  |  |  |  |  |  |  |
| *Mohan*  *2024* | Y | N | Y | Y | Y | Y | Y | Y | Y | Y | N | Y | Y | Y | Y | Y | Y | Y | Y | Y | 18/20 |
| *Riedhammer*  *2024* | Y | N | Y | Y | Y | Y | Y | Y | Y | Y | N | Y | Y | Y | Y | Y | Y | Y | Y | Y | 18/20 |
| *Al Hadidi*  *2025* | Y | N | Y | Y | Y | Y | Y | Y | Y | Y | N | Y | Y | Y | Y | Y | Y | Y | Y | Y | 18/20 |
| *Cani*  *2025* | Y | N | N | Y | Y | Y | Y | Y | Y | Y | N | Y | Y | Y | Y | Y | Y | Y | Y | Y | 17/20 |
| *Frenking*  *2025* | Y | N | Y | Y | Y | Y | Y | Y | Y | Y | N | Y | Y | Y | Y | Y | Y | Y | Y | Y | 18/20 |
| *Mian*  *2025* | Y | N | Y | Y | Y | Y | Y | Y | Y | Y | N | Y | Y | Y | Y | Y | Y | Y | Y | Y | 18/20 |
| *Razzo*  *2025* | Y | N | Y | Y | Y | Y | Y | Y | Y | Y | N | Y | Y | Y | Y | Y | Y | Y | Y | Y | 18/20 |
| *Sheu*  *2025* | Y | N | N | Y | Y | Y | Y | Y | Y | Y | N | Y | Y | Y | Y | Y | Y | Y | Y | Y | 17/20 |
| *Stork*  *2025* | Y | N | Y | Y | Y | Y | Y | Y | Y | Y | N | Y | Y | Y | Y | Y | Y | Y | Y | Y | 18/20 |
| *Tan*  *2025* | Y | N | Y | Y | Y | Y | Y | Y | Y | Y | N | Y | Y | Y | Y | Y | Y | Y | Y | Y | 18/20 |
| *Yi*  *2025* | Y | N | Y | Y | Y | Y | Y | Y | Y | Y | N | Y | Y | Y | Y | Y | Y | Y | Y | Y | 18/20 |
| ***CAR-T*** |  |  |  |  |  |  |  |  |  |  |  |  |  |  |  |  |  |  |  |  |  |
| *Logue*  *2022* | Y | N | Y | Y | Y | Y | Y | Y | Y | Y | N | Y | Y | Y | Y | Y | Y | Y | Y | Y | 18/20 |
| *Rejeski*  *2023* | Y | N | Y | Y | Y | Y | Y | Y | Y | Y | N | Y | Y | Y | Y | Y | Y | Y | Y | Y | 18/20 |
| *Dima*  *2024* | Y | N | Y | Y | Y | Y | Y | Y | Y | Y | N | Y | Y | Y | Y | Y | Y | Y | Y | Y | 18/20 |
| *Trando*  *2024* | Y | N | N | Y | Y | Y | Y | Y | Y | Y | N | Y | Y | Y | Y | Y | Y | Y | Y | Y | 17/20 |
| *Sidana*  *2025* | Y | N | Y | Y | Y | Y | Y | Y | Y | Y | N | Y | Y | Y | Y | Y | Y | Y | Y | Y | 18/20 |

Y = YES; N = NO; P = PARTIAL; U = UNCLEAR. A study with ≥ 70% of positive responses (12/18 or 14/20) was considered to be of acceptable quality.

Institute of Health Economics (IHE). Quality Appraisal of Case Series Studies Checklist. Edmonton (AB): Institute of Health Economics; 2014. Available from: <http://www.ihe.ca/research-programs/rmd/cssqac/cssqac-about>

References:

Moga C, Guo B, Schopflocher D, Harstall C. Development of a quality appraisal tool for case series studies using a modified Delphi technique. Edmonton: Institute of Health Economics; 2012. Available at <http://www.ihe.ca/advanced-search/development-of-a-quality-appraisal-tool-for-case-series-studies-> [using-a-modified-delphi-technique](http://www.ihe.ca/advanced-search/development-of-a-quality-appraisal-tool-for-case-series-studies-using-a-modified-delphi-technique) . Accessed January 8, 2016.

Guo B, Moga C, Harstall C, Schopflocher D. A principal component analysis is conducted for case series quality appraisal checklist. Journal of Clinical Epidemiology 2016;69:199-207.

S-Table 2. Summary of Findings Table.

1. Summary of findings table for BiTEs therapy.

| **Certainty assessment** | | | | | | | **№ of patients** | | **Effect** | | **Certainty** | **Importance** |
| --- | --- | --- | --- | --- | --- | --- | --- | --- | --- | --- | --- | --- |
| **№ of studies** | **Study design** | **Risk of bias** | **Inconsistency** | **Indirectness** | **Imprecision** | **Other considerations** | **Treated** | **Untreated** | **Relative (95% CI)** | **Absolute (95% CI)** |  |  |
| **Infection risk for BiTEs therapy (follow-up: mean 7.8 months; assessed with: proportion rate)** | | | | | | | | | | | | |
| 11^a^ | non-randomised studies | serious^b^ | not serious^c^ | not serious | not serious^d^ | publication bias suspected^e^ | 1,602 | -^f^ | - | **0.26**  (0.23 to  0.30) | ⨁⨁◯◯  Low | CRITICAL |

**CI:** confidence interval.

**Explanations**

a. All included studies scored above 70% on the IHE checklist, indicating good methodological quality.

b. All included studies were retrospective observational analyses. As such, they are subject to inherent limitations, including potential selection bias, incomplete outcome reporting, and variability in follow-up and infection ascertainment. However, the outcome (grade 3-4 infections) is relatively objective, which mitigates some risk. Overall, the risk of bias was considered serious**.**

c. Moderate heterogeneity was observed (I²=50.6%), indicating variability among study estimates. The direction of the effect was consistent across studies, and the confidence interval of the pooled proportion was relatively narrow (0.23-0.30). Therefore, inconsistency was judged not serious.

d. The pooled infection rate was 0.26 (95% CI 0.23-0.30), with a narrow confidence interval indicating a precise estimate of the proportion of patients experiencing infection. The sample size and number of events were sufficient to ensure statistical stability, and therefore imprecision is not considered serious.

e. Publication bias was assessed using a funnel plot of Freeman–Tukey transformed proportions and further tested with Egger’s regression. The funnel plot indicates a potential publication bias. The Egger's test supports the presence of funnel plot asymmetry (intercept: 3.14, 95% CI:1.66 - 4.62, t: 4.162, p-value: 0.002). The certainty of the evidence was downgraded by one level for suspected publication bias.

f. Control group are not considered in this meta-analysis.

1. Summary of findings table for CAR-T therapy.

| **Certainty assessment** | | | | | | | **№ of patients** | | **Effect** | | **Certainty** | **Importance** |
| --- | --- | --- | --- | --- | --- | --- | --- | --- | --- | --- | --- | --- |
| **№ of studies** | **Study design** | **Risk of bias** | **Inconsistency** | **Indirectness** | **Imprecision** | **Other considerations** | **Treated** | **Untreated** | **Relative (95% CI)** | **Absolute (95% CI)** |  |  |
| **Infection risk for CAR-T therapy (follow-up: mean 10 months; assessed with: proportion rate)** | | | | | | | | | | | | |
| 5^a^ | non-randomised studies | serious^b^ | serious^c^ | not serious | not serious^d^ | publication bias not suspected^e^ | 495 | -^f^ | - | **0.19**  (0.12 to  0.27) | ⨁⨁◯◯  Low | CRITICAL |

**CI:** confidence interval.

**Explanations**

a. All included studies scored above 70% on the IHE checklist, indicating good methodological quality.

b. All included studies were retrospective observational analyses. As such, they are subject to inherent limitations, including potential selection bias, incomplete outcome reporting, and variability in follow-up and infection ascertainment. However, the outcome (grade 3-4 infections) is relatively objective, which mitigates some risk. Overall, the risk of bias was considered serious**.**

c. High heterogeneity was observed (I²=70.9%), indicating high variability among study estimates. The direction of the effect was consistent across studies, and the confidence interval of the pooled proportion was relatively narrow (0.12-0.27). Therefore, inconsistency was judged serious.

d. The pooled infection rate was 0.19 (95% CI 0.12-0.27), with a narrow confidence interval indicating a precise estimate of the proportion of patients experiencing infection. The sample size and number of events were sufficient to ensure statistical stability, and therefore imprecision is not considered serious.

e. Publication bias was assessed using a funnel plot. The funnel plot does not indicate a potential publication bias. The Egger's test does not support the presence of funnel plot asymmetry (intercept: 2.54, 95% CI:-1.93 - 7.01, t: 1.113, p-value: 0.347).

f. Control group are not considered in this meta-analysis.

S-Table 3. Studies’ characteristics at baseline.

| **study,**  ***year*** | **study**  **type** | **treatment** | **patients at baseline, *n°*** | **treatment duration, months (median)** | **DOI** |
| --- | --- | --- | --- | --- | --- |
| Mohan, 2024 | R, MC | teclistamab | 110 | 3.5 | 10.1038/s41408-024-01003-z |
| Riedhammer, 2024 | R, MC | teclistamab | 123 | 6 | 10.1038/s41375-024-02154-5 |
| Al Hadidi, 2025 | R, MC | talquetamab | 114 | 6 | 10.1038/s41408-025-01386-7 |
| Cani, 2025 | R, SC | BiTEs | 158 | 5.5 | 10.3324/haematol.2025.288187 |
| Frenking, 2025 | R, MC | talquetamab | 138 | 7.8 | 10.1002/hem3.70114 |
| Mian, 2025 | R, MC | teclistamab | 81 | 13.1 | 10.1038/s41375-025-02552-3 |
| Razzo, 2025 | R, MC | teclistamab | 509 | 10.1 | 10.1158/2643-3230.BCD-24-0354 |
| Sheu, 2025 | R, SC | teclistamab | 44 | 6.7 | 10.1159/000548964 |
| Stork, 2025 | R, MC | teclistamab | 73 | 4.9 | 10.1007/s00277-025-06529-1 |
| Tan, 2025 | R, MC | teclistamab | 210 | 5.3 | 10.1038/s41408-025-01259-z |
| Yi, 2025 | R, MC | teclistamab | 42 | 16.4 | 10.4143/crt.2025.399 |
| Logue, 2022 | R, MC | ide-cel | 52 | 3 | 10.1182/bloodadvances.2022008320 |
| Rejeski, 2023 | R, MC | BCMA directed CAR-T | 113 | 7.9 | 10.1186/s13045-023-01465-x |
| Dima, 2024 | R, MC | idecel | 69 | 10 | 10.1111/bjh.19302 |
| Trando, 2024 | R, SC | idecel | 25 | 6 | 10.3390/biomedicines13010036 |
| Sidana, 2025 | R, MC | ciltacel | 236 | 13 | 10.1182/blood.2024025945 |
| **total** |  |  | **2,097** | **8.3^*^** |  |

MC, multicenter; R, retrospective; SC, single center,

^*^Value represents the weighted mean of the medians reported in individual studies, weighted by the number of patients in each trial.

S-Table 4. Patient’s characteristics at baseline.

| **study,**  ***year*** | **n° patients** | **age,**  ***mean*** | **female,**  ***n°* (%)** | **n° previous line of treatment, mean** | **previous ASCT, %** | **EMD, %** | **high cytogenetic risk, %** | **ISS III, %** | **ISS I, %** | **BCMA exposed, %** | **pentarefractory, %** |
| --- | --- | --- | --- | --- | --- | --- | --- | --- | --- | --- | --- |
| Mohan, 2024 | 110 | 67.5 | 54 (49) | 6.2 | 87 | 44 | 62* | n.a. | n.a. | 35 | 76 |
| Riedhammer, 2024 | 123 | 66.4 | 53 (43.1) | 6.2 | n.a. | 36.1* | 36.8* | 33.7* | 27.1* | 37.4 | 60.2 |
| Al Hadidi, 2025 | 114 | 66.5 | 50 (44) | 6.3 | 81.6 | 34* | 70* | n.a. | n.a. | 64.9 | 78.9 |
| Cani, 2025 | 158 | 65.5 | n.a. | 5.8 | 81 | n.a. | n.a. | n.a. | n.a. | 41 | n.a. |
| Frenking, 2025 | 138 | 63.1 | 42 (30) | 6.2 | 86 | 48* | 48* | 43* | 30* | 51 | 47* |
| Mian, 2025 | 81 | 76.6 | 33 (40.7) | 6.7 | n.a. | 38.7* | 56.9* | 32* | 28* | 38.3 | 46* |
| Razzo, 2025 | 509 | 67.8 | 234 (46) | 6.1 | 65.5*^#^ | 44.7* | 54 | n.a. | n.a. | 46.4 | 38* |
| Sheu, 2025 | 44 | 67.8 | 20 (45.5) | 6.9 | 6.8 | n.a. | 50 | n.a. | n.a. | 45.5 | n.a. |
| Stork, 2025 | 73 | 66.1 | 37 (50.7) | 5.3 | n.a. | 24.7 | 44* | 26 | 30.1 | 12.3 | 68.5 |
| Tan, 2025 | 210 | 66.7 | 93 (44.3) | 6.3 | n.a. | 29.4* | 50* | 30.6* | 37.9* | 43.8 | 44.1* |
| Yi, 2025 | 42 | 66.8 | 15 (35.7) | 6.1 | 86 | 28.6 | 42.9 | 40.5 | 21.4 | 7.1 | 19 |
| Logue, 2022 | 52 | 65.0 | 29 (56) | 6.4 | 81 | 62 | 40 | n.a. | n.a. | 10 | 44 |
| Rejeski, 2023 | 113 | 64.2 | 48 (42) | 5.9 | 88 | 45.1 | 29.5* | n.a. | n.a. | 13* | 42.5 |
| Dima, 2024 | 69 | 63.4 | 35 (51) | 6.5 | n.a. | 52 | 40* | n.a. | n.a. | 26 | 48 |
| Trando, 2024 | 25 | 64.9 | 13 (52) | 6.5 | 100 | 40 | 48 | n.a. | n.a. | 16 | 88 |
| Sidana, 2025 | 236 | 63.0 | 102 (43) | 6.0 | 85 | 25.5* | 40.7* | n.a. | n.a. | 14 | 30 |
| **total** | **2,097** | **66.3** | **858 (44.2)** | **6.1** | **77.4^§^** | **39.2^§^** | **48.8^§^** | **34.3^§^** | **30.5^§^** | **37.0^§^** | **47.1^§^** |

​​n.a., not available.

^#^This value includes both autologous SCT and allogeneic STC.

^*^Percentage is calculated based on the total of patients assessed for that clinical manifestation.

^§^The denominator does not include patients that were not assessed for that clinical manifestation.

S-Figure 2. Meta-regression analysis for percentage of BCMA exposed patients prior BiTEs therapy.

Meta-regression analysis for percentage of BCMA exposed patients for each study on infection rate (%).

S-Table 5. Severe infection rate, BiTEs and CAR-T-related toxicities, overall remission rate and substitutive immunoglobulins use across the studies.

| **study,**  ***year*** | **n° patients** | **patients with Grade 3-4 infection,**  ***n°* (%)** | **CRS all grade, %** | **ICANS, %** | **ORR, %** | **IgRT, %** |
| --- | --- | --- | --- | --- | --- | --- |
| Mohan, 2024 | 110 | 29 (26) | 56 | 11 | 62 | 43 |
| Riedhammer, 2024 | 123 | 33 (26.8) | 58.5 | 7.3 | 59.3 | n.a. |
| Al Hadidi, 2025 | 114 | 20 (17.5) | 54* | 9.8* | 73* | 61 |
| Cani, 2025 | 158 | 39 (24.7) | 25 | 11 | n.a. | 70* |
| Frenking, 2025 | 138 | 37 (28.2) | 70* | 9* | 65 | 50 |
| Mian, 2025 | 81 | 19 (23.4) | 49.4 | 16.9 | 63* | n.a. |
| Razzo, 2025 | 509 | 113 (22) | 54 | 11 | 53* | 38 |
| Sheu, 2025 | 44 | 17 (38.6) | n.a. | n.a. | n.a. | 65.2 |
| Stork, 2025 | 73 | 26 (35.6) | 47.9 | 2.7 | 58.8* | 75.3 |
| Tan, 2025 | 210 | 46 (21.9) | 54 | 7.3* | 67 | 62* |
| Yi, 2025 | 42 | 18 (42.9) | 47.6 | 0 | 66.7 | 50 |
| Logue, 2022 | 52 | 12 (23) | 85 | 20 | 92 | 13 |
| Rejeski, 2023 | 113 | 23 (20.4) | n.a. | 20.4 | 85.2 | 21.2 |
| Dima, 2024 | 69 | 21 (30) | 81 | 28 | 93 | n.a. |
| Trando, 2024 | 25 | 3 (12) | 92 | 12 | 84 | 80 |
| Sidana, 2025 | 236 | 49 (20.8) | 75.3* | 13.9* | 89 | n.a. |
| **total** | **2,097** | **505 (24.2)^§^** | **57.5^§^** | **11.6^§^** | **67.8^§^** | **48.4^§^** |

CRS, cytokine release syndrome; ICANS, immune effector cell-associated neurotoxicity syndrome; IgRT, immunoglobulin replacement treatment; n.a., not available; ORR, overall response rate.

*Percentage is calculated based on total of patients assessed for that clinical manifestation.

^§^The denominator does not include patients that were not assessed for that clinical manifestation.

S-Table 6. Comparison between meta-analysis on severe infection rate.

|  | **current study** | **Reynolds et al.** | **Wang et al.** | **Li et al.** | **Bakogeorgou et al** | **Techaapornkun et al.** | **Vandenboom et al.** |
| --- | --- | --- | --- | --- | --- | --- | --- |
| **type of studies included** | real-life | trials | trials | trials, real-life | trials | real-life | trials |
| **also includes investigational BiTEs or CAR-T, Y/N** | N | Y | Y | N | Y | Y | Y |
| **BCMA BiTEs severe infection rate** | 27% | n.a. | 25% | 50%*; 24%^Ψ^ | n.a. | n.a. | n.a. |
| **non-BCMA BiTEs severe infection rate** | 25% | n.a. | 20% | n.a. | n.a. | n.a. | n.a. |
| **GPR5CD BiTEs severe infection rate** | 25% | n.a. | n.a. | n.a. | n.a. | n.a. | n.a. |
| **overall BiTEs severe infection rate** | 26% | 21% | 22% | 50%*; 24%^Ψ^ | 29% | 39% | 30% |
| **CAR-T severe infection rate** | 17% | n.a. | n.a. | n.a. | n.a. | 25% | 17% |

N, not; Y, yes.

*clinical trials.

^Ψ^real-life studies.
